# Rehashing Our Insight of Seaweeds as a Potential Source of Foods, Nutraceuticals, and Pharmaceuticals

**DOI:** 10.3390/foods12193642

**Published:** 2023-10-01

**Authors:** Ravi S. Baghel, Babita Choudhary, Sonika Pandey, Pradeep Kumar Pathak, Manish Kumar Patel, Avinash Mishra

**Affiliations:** 1 Biological Oceanography Division, CSIR-National Institute of Oceanography, Panaji 403004, Goa, India; rsbaghel@nio.org; 2 Division of Applied Phycology and Biotechnology, CSIR, Central Salt and Marine Chemicals Research Institute, G. B. Marg, Bhavnagar 364002, Gujarat, India; choudharybabita212@gmail.com; 3 Academy of Scientific and Innovative Research (AcSIR), Ghaziabad 201002, Uttar Pradesh, India; 4 Department of Fruit Tree Sciences, Institute of Plant Sciences, Agricultural Research Organization, Volcani Center, Rishon LeZion 7528809, Israel; sonikapandey14@gmail.com; 5 Department of Postharvest Science of Fresh Produce, Agricultural Research Organization (ARO), Volcani Center, Rishon LeZion 7505101, Israel; pkpathak21@gmail.com

**Keywords:** biologically active compounds, food additives, functional foods, nutraceuticals and pharmaceuticals, polysaccharides, secondary metabolites, seaweeds

## Abstract

In a few Southeast Asian nations, seaweeds have been a staple of the cuisine since prehistoric times. Seaweeds are currently becoming more and more popular around the world due to their superior nutritional value and medicinal properties. This is because of rising seaweed production on a global scale and substantial research on their composition and bioactivities over the past 20 years. By reviewing several articles in the literature, this review aimed to provide comprehensive information about the primary and secondary metabolites and various classes of bioactive compounds, such as polysaccharides, polyphenols, proteins, and essential fatty acids, along with their bioactivities, in a single article. This review also highlights the potential of seaweeds in the development of nutraceuticals, with a particular focus on their ability to enhance human health and overall well-being. In addition, we discuss the challenges and potential opportunities associated with the advancement of pharmaceuticals and nutraceuticals derived from seaweeds, as well as their incorporation into different industrial sectors. Furthermore, we find that many bioactive constituents found in seaweeds have demonstrated potential in terms of different therapeutic attributes, including antioxidative, anti-inflammatory, anticancer, and other properties. In conclusion, seaweed-based bioactive compounds have a huge potential to play an important role in the food, nutraceutical, and pharmaceutical sectors. However, future research should pay more attention to developing efficient techniques for the extraction and purification of compounds as well as their toxicity analysis, clinical efficacy, mode of action, and interactions with regular diets.

## 1. Introduction

The demand for food, chemicals, and energy is rising along with the world’s population growth. Since land resources cannot be fully utilized for agriculture, the world is now turning its attention to the potential of the ocean to meet these needs [1]. At the same time, seaweeds (macroalgae) are attracting a lot of attention due to their distinctive qualities, including their widespread occurrence, enormous availability, high production rate, and lack of agricultural inputs when grown using cutting-edge cultivation techniques. Based on their pigmentation, seaweeds (macroalgae) are generally classified as Chlorophyta, Rhodophyta, and Phaeophyta [2,3]. Green seaweeds, called chlorophyta, have chlorophyll a and b as their principal pigments. Red seaweeds belonging to the Rhodophyta class predominately contain phycobiliprotein. The third is Phaeophyta, a type of brown seaweed that predominately includes fucoxanthin. Being aquatic organisms, seaweeds have a large percentage of water in their bodies—up to 85% [4]—with organic matter and minerals making up the majority of the rest of the body. Seaweed dry matter is composed of 0.5–3.5% lipids, 21–61% carbohydrates, 3–50% proteins, and 12–46% minerals [5,6,7,8]. 

Many nations, including Costa Rica, Japan, China, and Egypt, have used specific seaweeds rich in protein and vitamins, such as the red seaweed *Porphyra* (Nori), as food since prehistoric times [1,9,10,11]. According to Baghel et al. (2017) [12], the industrial manufacture of hydrocolloids like agar, agarose, carrageenan, and alginate has long been the principal usage of seaweed biomass. These hydrocolloids are widely used as ingredients in a variety of industries, including those that deal with food, drinks, dairy, cosmetics, personal care, pharmaceuticals, healthcare, textiles, printing, and paper coating [12]. Three categories of seaweed that produce hydrocolloids are recognized. Seaweeds containing agar are referred to as agarophytes, those that contain carrageenan are termed carrageenophytes, and those that contain alginate are termed alginophytes [13]. Global hydrocolloid production was estimated in 2015 at roughly 120,000 tonnes [14]. Additionally, considerable levels of storage sulfated polysaccharides like ulvan and porphyran are present in members of the genera *Ulva* and *Porphyra*. Such polysaccharides have the potential to be used to make medicines and nutraceuticals due to their wide range of biological activities [15]. The abundance of algae taxa and their diverse chemical makeup offer numerous options for the bio-prospecting of this precious resource. Cai et al. (2021) [16] calculated that 35.8 million wet tonnes of seaweed were harvested in 2019. The composition of seaweed differs between species as well as within groups [17,18]. As a result, many seaweed extracts exhibit a wide range of bioactivities, including anti-inflammatory, antiviral, antifungal, anticancer, and neuroprotective properties. Numerous studies that highlight the biological functions of extracts from seaweed have been published. This review was conducted in order to provide a comprehensive description of seaweed compounds and their biological activities. Most of the information was collected from articles published in the past two decades. The review starts with a general overview of seaweeds and their applications. The manuscript discusses bioactive compounds and their biological properties. Comprehensive accounts of the principal and secondary seaweed-based metabolites are covered in this review. Additionally, examples are provided for the description of biological activities such as neuroprotective, anti-tumor, antifungal, antiviral, anti-coagulant, and antioxidant properties.

## 2. Seaweeds as a Source of Nutrients and Metabolites

**Figure 1:**
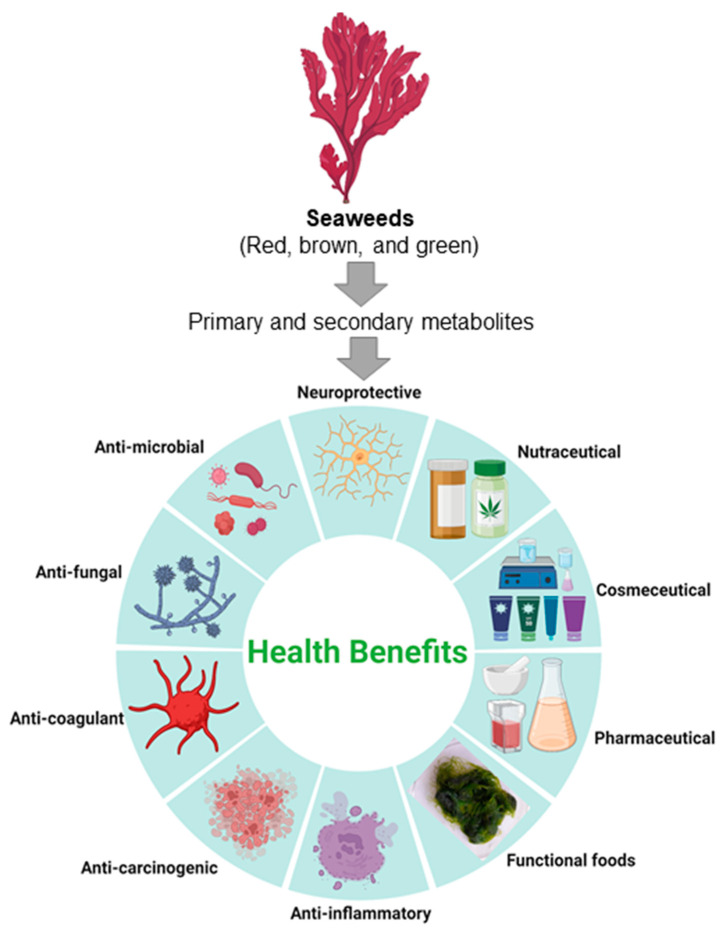
A schematic illustration of seaweed primary and secondary metabolites and their possible application as bioactive compounds and functional foods with the desired benefits.

A growing population with a high risk of health issues necessitates the development of functional foods that can address food security issues while also providing a rich source of bioactive compounds. Seaweeds have been consumed in East Asian areas since ancient times, and in recent years, seaweeds have piqued the interest of researchers due to their natural source of bioactive compounds with the potential to be nutraceuticals (Figure 1) [19]. Seaweeds are a rich source of macrominerals (K, Mg, Ca, and P) and microminerals (Fe, Mn, Mo, Ni, Se, and Zn) (Table 1), along with vitamins, proteins, and carbohydrates [20,21]. In addition, when compared to other vegetables, they have high levels of essential amino acids and unsaturated fatty acids [21]. The field of metabolomics, a branch of “omics” study, offers insights into the primary and secondary metabolites of a certain organism at a specific time [22]. It is well known that seaweed extracts contain a variety of other metabolites, including secondary metabolites, and have known bioactive properties [23]. Because they may produce a number of secondary metabolites that shield them from structural and photodynamic damage, seaweeds can grow luxuriantly [24]. Secondary metabolites are predominantly produced in response to various stress conditions, such as exposure to ultraviolet (UV) radiation, temperature, salinity stress, or environmental pollutants. As a result of the algae’s exposure to these conditions during mariculture, these metabolites are present in significant amounts in seaweeds. Among other bioactive compounds, polyphenols, sterols, halogenated compounds, terpenes, and small peptides are secondary metabolites algae produce [23]. Currently, seaweeds are gaining popularity as a natural source of bioactive substances that play a vital role in the creation of nutraceuticals. In terms of species, habitat, age, environmental factors, and time of harvest, seaweeds are incredibly diverse, yet they are great suppliers of many nutrients [24]. Algal extracts from *Kappaphycus alvarezii* and other seaweeds have been reported to have various phytochemicals like flavonoids, saponins, steroids, and squinones [23]. It is advised to use seaweed flour as an alternative to conventional flour since it contains polysaccharides, protein, and dietary fiber, which are natural antioxidants with high nutritional value [25].

### 2.1. Micronutrient Composition

#### 2.1.1. Minerals

In humans, dietary minerals are essential components of food and are needed to drive different cellular, physiological, and metabolic activities inside the cell. Since minerals cannot be synthesized inside the body, they need to be supplied through food intake. Minerals are broadly classified into two groups based on daily body requirements. Minerals required at >100 mg/day are called macrominerals, while those required at less than 100 mg/day are known as trace minerals. Sodium, calcium, magnesium, potassium, chloride, phosphorus, and sulfur are major minerals used in large quantities in the body [36]. Trace minerals are just as vital to our health as major minerals, but we do not need large amounts. Minerals in this category include chromium, copper, fluoride, iodine, iron, manganese, molybdenum, selenium, and zinc. Apart from these essential minerals, toxic minerals such as Hg, Pb, Cd, As [37,38], and Al [37] are also present in seaweeds and can have a detrimental effect on the human body by impairing different chemical reactions.

Seaweeds are considered a superfood and can be used in dietary supplements to find solutions for mineral deficiency and human diseases. Compared to land plants, the mineral content of many seaweeds has been reported to be 40% of the dry weight, and one gram of dried seaweed can provide ten times the mineral content when consumed [39,40]. However, the chemical composition of minerals in seaweeds is highly variable and is defined by the species type, geographical distribution, season changes, time of harvesting, and many other environmental factors such as intensity of light, water temperature, salinity, and availability of nutrients in the water [41,42]. Seaweeds have been found to have outstanding sorption capabilities for a variety of inorganic ions from their habitat, such as seawater. One such method is called “biosorption”, which uses the capacity of microbial and plant biomass to physically and chemically sequester heavy metal ions from water. In addition, the cell walls of seaweeds are composed of complex polysaccharides consisting of polymers differing in monosaccharide composition, glycosidic linkages, configuration, molecular mass, and functional groups. Based on polysaccharides, seaweeds are characterized by three taxa, namely red algae consisting of sulfated galactans (agars and carrageenans), brown algae formed of alginates and fucoidans, and green algae made up of sulfated glucuronoxylorhamnans (ulvans) and other sulfated glycans [43,44]. Recent research has revealed that the sequestration of metal ions is thought to be caused by charged groups like carboxylate and hydroxyl in the biopolymers of biomass cell walls [45,46]. Additionally, the makeup of different seaweed species’ cell walls significantly determines how well they absorb and assimilate minerals. Previous studies have well documented that brown seaweeds have a higher capacity for mineral absorption than green and red seaweeds, which is due to the high presence of alginic acid, alginate, and the salts of alginic acids. For example, in brown seaweeds such as *Undaria pinnatifida*, *Fucus vesiculosus*, and *Laminaria digitata* and in red seaweeds such as *Porphyra tenera*, *Gracilaria corticata*, *G*. *dura*, and *Chondrus crispus*, the mineral contents have been investigated using atomic absorption spectrophotometry. Seaweeds are known to contain as much as 21.1–39.3% ash and 1.3–5.9% sulfate [20,27]. An edible seaweed, *Kappaphycus alvarezzi*, was reported to be a rich source of different minerals, such as calcium (0.16%), iron (0.033%), and zinc (0.016%) [47]. In addition, *Eisenia arborea*, a brown alga commonly found along the western coast of the Baja California Peninsula of Mexico, contains the four minerals K, Na, Mg, and Ca, ranging from 907 to 7946 mg/100 g [48]. A diet with low Na^+^/K^+^ is generally considered healthy because high Na+ intake can lead to hypertension [49,50]. Several species of red algae, such as *Gracilaria corticata*, *Acanthophora spicifera*, and *G. pudumadensis*, are reported to contain high K [40,51]. In addition the brown algae *Macrocystis integerifolia* and *Nereocystis luetkeana* are also known to contain high K and low Na, whereas green seaweeds are recorded to contain high Na and low K [51,52]. Nutrient supplements made from algal seaweeds would also provide some trace elements for adults, namely Fe, 10–18 mg; Zn, 15 mg; Mn, 2.5–5 mg; and Cu, 2–3 mg [53]. Among the green seaweeds, *Caulerpa racemosa* and *Caulerpa lentilifera* were reported to have higher Na (10–16%), followed by 2.2–4% K, 0.46–0.52% Ca, and 0.27–0.33% P [54] as well as microminerals: 386–756 ppm Fe, 59–519 ppm Mn, and 3.3–10.0 ppm Zn [54]. Seaweeds such as *S*. *wightii* (0.39% to 0.49% dw) and *C*. *minima* (2.71 mg/100 g dry wt) are also known sources of iron (Fe), which is an important part of hemoglobin and is required in the amounts of 10–12 mg daily for men and 15 mg daily for women.

Iodine is one of the essential nutrients for health, and some seaweeds have iodine concentrations that are higher than the daily recommended intake of 150 mg for humans [55]. Red and green seaweeds have lower iodine contents than brown seaweeds, especially kelps, which have the highest concentration [55,56]. In addition, brown algae from the order Laminariales are the highest accumulators of iodine [57] and can accumulate iodine to more than 30,000 times the concentration of this element in seawater, which is mainly due to the presence of haloperoxidases in the cell wall. This enzyme is reported to catalyze the oxidation of iodide by hydrogen peroxide, thus facilitating the absorption and storage of iodine inside the cell [58]. In seawater, iodine is mainly present as I^−^ and IO3. Moreover, exposure to oxidative stress leads to the conversion and release of high levels of iodide. Through microimaging, it has been revealed that iodine is mainly localized in the extracellular matrix of cells located in the peripheral tissues [57]. Many commercially available red algae, such as *C*. *crispus* and *M*. *stellatus*, are also reported to have high iodine contents, very similar to a few brown species, including *Fucales* [59]. Thus, because seaweeds contain a high amount of iodine, they can act as a natural source of iodine nutraceuticals. Some commonly available seaweeds that are good sources of iodine are the brown seaweeds belonging to the genera *Laminaria*, *Undaria*, *Sargassum*, *Macrocystis*, *Ecklonia*, *Durvillaea*, and *Ascophylum* [60]; red seaweeds of the genera *Palmaria*, *Gracilaria*, *Gelidium*, *Laurencia*, and *Chondrus*; and green seaweeds of genera *Enteromorpha*, *Codium*, *Ulva,* and *Monostroma*.

#### 2.1.2. Vitamins

Vitamins are micronutrients that serve multiple and unique functions inside the cell, such as growth and promoting body cell development [61]. In addition, some vitamins also act as co-enzymes for enzymatic reactions. A deficiency of vitamins in humans not only hampers the normal functioning of the body but also causes severe vitamin deficiency. Therefore, a regular intake of vitamins through food is essential. Seaweeds are natural sources of hydrosoluble (B1, B2, B12, and C) and liposoluble vitamins (thiamine, riboflavin, b-carotene, and tocopherols). Vitamin C (L-ascorbic acid) is essential for strengthening of the immune system and iron absorption in the human body. A deficiency of this vitamin can lead to scurvy. On average, green and brown algae can provide 500 to 3000 mg/kg of dry matter of Vitamin C, whereas red algae contains Vitamin C levels of around 100 to 800 mg/kg [41,62]. Vitamin C is reported to be highest in green seaweeds, ranging from 50–300 mg, and lowest in red algae, at 10–80 mg 100 g^−1^ [48,56]. *Ulva lactuca* (sea lettuce), *Undaria pinnatifida* (Wakame), *Gracilaria* spp. [63], and *Porphyra umbilicalis* are some seaweeds that contain a good amount of Vitamin C [33].

Seaweeds can be a major source of vitamins for vegetarians, since a large proportion of vitamins are of animal origin. Approximately 100 g of seaweed can provide more than the daily Vitamin A, B2, and B12 requirements and two-thirds of the Vitamin C requirement [64]. Like minerals, the composition and, therefore, the vitamin profile of seaweeds vary with algal species, growth stages, geographic area, salinity, season, availability of light, and temperature [63,65]. Light is frequently a crucial regulator of vitamin production; hence, plants grown in bright light have a higher ascorbate content [66]. Additionally, algae gathered from depths between 9 and 18 m are likely to have a higher level of Vitamin C than algae growing in the intertidal zone [67]. Additionally, other environmental factors, such as the concentration of particular substances in the sea, might significantly impact the presence of vitamins in algae [66]. Among the seaweed types, red seaweeds such as *Paimaria palmata* and *Porphyra tenera* are known to contain large amounts of provitamin A and significant quantities of Vitamins B1 and B2. Brown seaweeds are reported to have all forms of α, β, and γ-tocopherols, while green and red algae only contain α-tocopherol [62]. Moreover, the content of α-tocopherol is much higher in brown seaweeds when compared with red and green [68]. Vitamin E modulates prostaglandin production and inhibits LDL oxidation, thus having antioxidative and anti-inflammatory properties [62]. Vitamin B12 insufficiency is prevalent among those following strict vegetarian diets and plays a crucial role in several enzymatic processes occurring inside the human body. A deficiency of B12 can lead to anemia, chronic fatigue syndrome (CFS), and various skin issues. However, macroalgae, such as *Ulva lactuca*, are reported to be a good source of Vitamin B12 [69]. Consuming seaweed (approximately 100 g per day) provides more than the recommended daily amounts of Vitamins A, B2, and B12 and two-thirds of the recommended daily amount of Vitamin C [70].

### 2.2. Macronutrient Composition

Seaweeds have a high nutritional component compared to terrestrial plants because they do not contain the root, stem, leaves, or circulatory system, so their energy is not consumed [71]. In the human diet, edible seaweeds add macronutrients and micronutrients, especially in regions where seaweeds are used as a portion of regular food, such as Japan, where one-fifth of the population includes seaweeds in their food [72]. Laminarin, floridoside, cellulose, hemicelluloses, starch, and hydrocolloids (carrageenan, alginate, and agar) are among the polysaccharides found in seaweeds. Other components include different proteins, Vitamins A, B1, B2, B12, C, D, E, and K, as well as minerals like Mg, Ca, Fe, Na, K, I, F, Se, and Zn. Along with polyphenols, Vitamins C and E, carotenoids, phlorotannins, carotene, sterols, protein, catechins, and sulfated polysaccharides, seaweed also contains antioxidants. These fats are mostly in the form of mono- and polyunsaturated fatty acids, which are low in calories [73].

#### 2.2.1. Seaweed Polysaccharides

Carbohydrates are a major component of seaweeds, comprising approximately 50% of the dry weight. Polysaccharides are one of the earliest biopolymers among the numerous natural polymers. These are high-molecular-weight polymers and are found in all living things, including plants and seaweed. They are incredibly important for the survival of both plants and animals. Most polysaccharides have hydroxyl functional groups, making it simple to synthesize their chemical derivatives. Countless industrial applications utilize a variety of polysaccharide derivatives. They serve as providers of water and energy, along with structural materials. Due to their diverse structural and morphological plasticity, they are used in various sectors, from the food industry to the pharmaceutical industry. Seaweed polysaccharides swell in water and are soluble in hot water, resulting in colloidal, very viscous solutions or dispersions with pseudoplastic flow behavior. Therefore, they are advantageous for utilization in numerous applications as compared to polysaccharides derived from terrestrial biomass, such as cellulose, starch, and chitosan, among others [74]. Polysaccharides are beneficial in various applications due to their intrinsic functional qualities, which include thickening, water retaining and binding, stabilizing suspensions and emulsions, and gelling [75].

Polysaccharides can be extracted from seaweed in a variety of ways, including hot water extraction, ultrasonic-assisted extraction (UAE), microwave-assisted extraction, and enzymatic-assisted extraction [76,77]. Many in vivo and in vitro investigations have revealed that the intestinal gut microbiota substantially impacts host health and can be particularly altered by compounds usually referred to as prebiotics. The enzymes found in the upper part of the gastrointestinal tract do not break down the oligosaccharides and polysaccharides that marine macroalgae and microalgae produce [78]. Extracted algal polysaccharides have tremendous potential for the effective use of emergent prebiotics. They can also be added to feed or consumed as tablets [79]. Bio-based polymers are made from polysaccharides that are non-toxic and biodegradable. Additionally, these biological materials are well-known for being biocompatible, which is defined as “the ability of a material to perform with an appropriate host response in a specific application”. As a result, these traits provide important benefits for food packaging, especially for edible coatings and films. Polysaccharides also offer a sustainable choice for active packaging because of their innate antibacterial, antioxidant, and various other biological capabilities [80]. The physical characteristics, chemical makeup, ability to be fermented by the intestinal flora, and biological effects on animal and human cells of dietary fibers are all quite different. In comparison to higher plants, edible seaweed has a greater total fiber content of 33–50% on a dry weight basis, and these fibers are rich in soluble fractions. Agars, alginic acid, furonan, laminarin, and porphyran are examples of water-soluble dietary fibers, while cellulose, mannans, and xylan are examples of insoluble dietary fibers found in marine algae [81]. Seaweed-based polysaccharides are widely investigated for their biological activities (described in the biological activity of seaweeds section).

##### Sulfated Fucose-Fucoidans

The term “fucoidans” refers to a class of fucose-rich, sulfated polysaccharides typically composed of a backbone of variously substituted, l-fucose residues. Most fucoidan polysaccharides are discovered in brown seaweed, but different kinds of brown seaweed have different fucoidan structures. Fucoidans, a family of fucose-containing sulfated polysaccharides, are a group of chemically diverse structural entities (FCSPs) [82]. Fucoidan polysaccharides have various biological features with the potential for pharmaceutical applications, such as immunomodulatory, anti-tumor, anti-inflammatory, and anti-coagulant properties. The molecular weight of fucoidans, their sulfate content, the position of sulfate ester groups, and their monosaccharide composition all impact their bioactivities [83]. The fucoidan polysaccharides’ structural integrity and biological characteristics must be maintained by using a mild extraction method [84].

**Figure 2:**
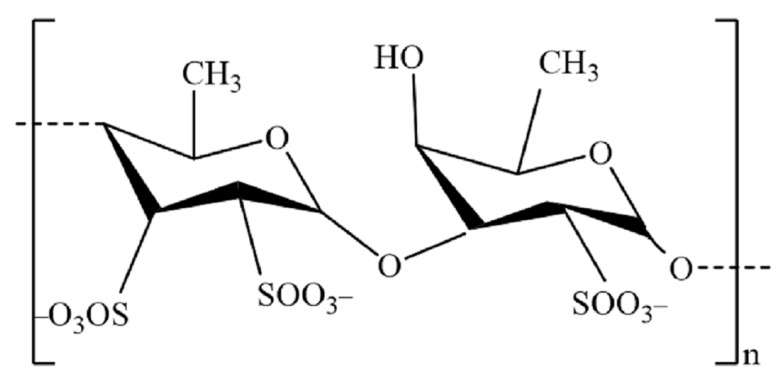
Fucoidan’s primary structure.

Fucoidans from brown seaweed typically have a backbone composed of l-fucopyranose residues or alternate l-fucopyranosyls linked by sulfate or acetate, and/or they may have side branches made of l-fucopyranoses or other glycosyl units, like glucuronic acid (Figure 2). Small amounts of several additional monosaccharides, such as glucose, galactose, xylose, and/or mannose, have also been found in a number of the fucoidan structures. Other polysaccharides could contaminate these monosaccharides or replace fucoidan’s molecular components [82,83]. A carbohydrate chain purified from *S*. *siliquosum* is composed of (13)- or (14)-linked l-fucose residues with sulfate groups at the C-2 and C-4 positions. The branch points, which are galactose residues with (1–4)-linkages, are situated at the C-3 or C-4 position of the fucose residues. While minor sulfation can also be found at C-2, galactose residues are primarily sulfated at C-4 and C-6. These purified substances displayed anti-inflammatory, antioxidant, and anti-lipogenic properties [85]. Japanese *Cladosiphon okamuranus* fucoidan seemed to have a fucose composition of 70.13 ± 0.22 wt% and a sulfate content of 15.16 ± 1.17 wt%. D-galactose, d-xylose, d-rhamnose, d-mannose, d-arabinose, d-glucose, and d-glucuronic acid were also identified as minor monosaccharides. According to linkage studies, the fucopyranoside units are connected to one another throughout the backbone by -1,3-glycosidic linkages, with fucose branching at C-2 and one sulfate group for every three fucose units at C-4, which shows fucoidan structure as [→3)-α-fuc(1→]_0.52_[→3)-α-fuc-4-OSO_3_-(1→]_0.33_[→2)-α-fuc]_0.14_ [86].

Variation in the fucoidan sulfate groups is crucial to the polysaccharide’s functioning. It has been suggested that the structure of fucoidan varies with species, season, location, and maturity. Industrial applications need to take into account fucoidan’s seasonal changes to ensure ideal harvesting times and maintain a consistent product composition. In a report, brown seaweeds *Fucus vesiculosus*, *Fucus serratus*, and *Ascophyllum nodosum* were studied, and the findings revealed considerable variations in the extracted fucoidan quantity and structure depending on the species and the month [87].

##### Sulfated Galactan-Carrageenans

**Figure 3:**
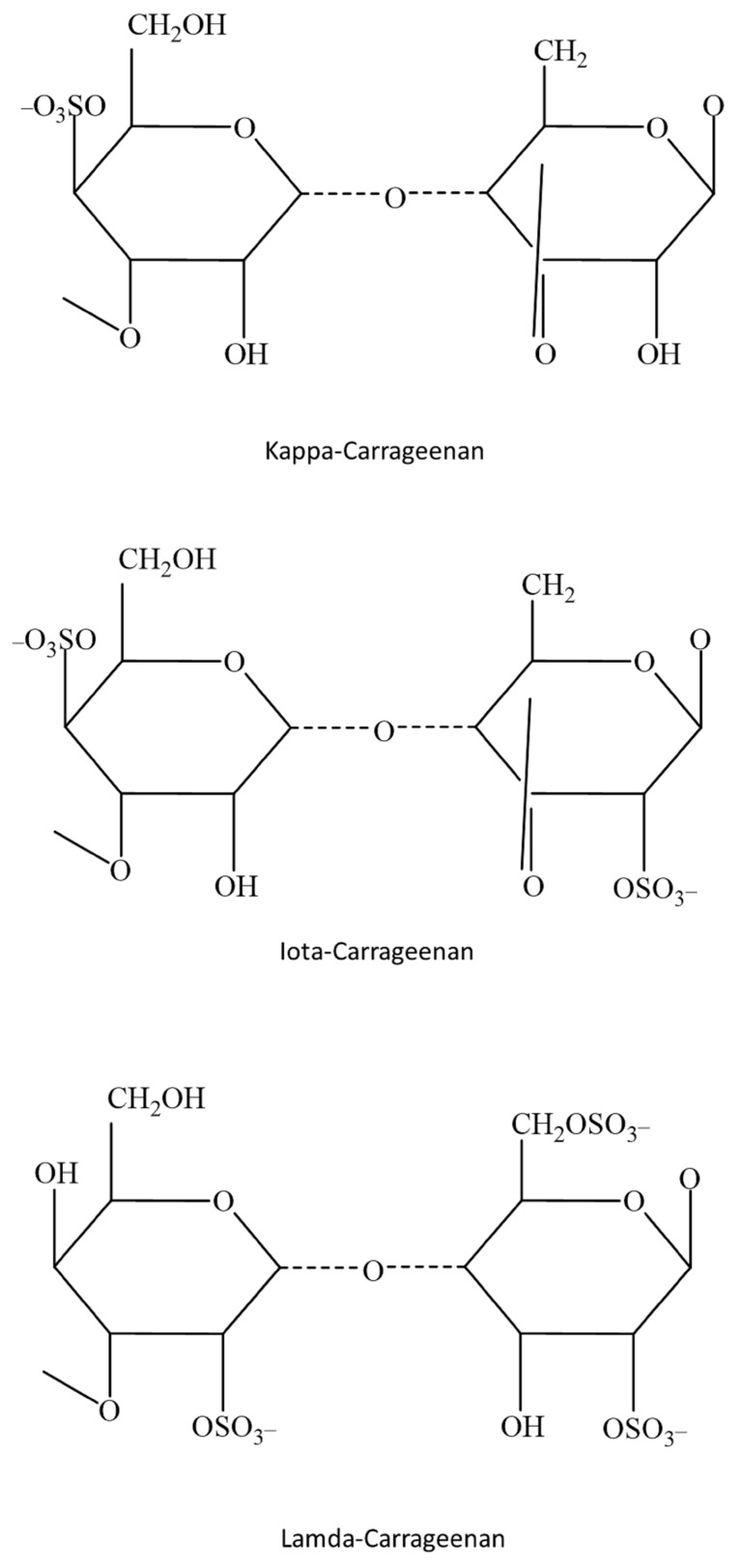
Structure of different carrageenans.

Agarans and carrageenans comprise 40–50% of the dry weight of red seaweed and are the primary cell wall polysaccharides. These are crucial for creating foods, medicines, and cosmetics due to their biocompatibility, biodegradability, and low toxicity [88]. Carrageenans are made up of [→3)-β-d-Gal*p*-(1→4)-α-d-Gal*p*-(1→] (Figure 3), also known as “diads” or “carrabiose”, and are frequently substituted by sulfate groups and infrequently by other substituents [89]. According to the position of sulfate groups in the β-galactose moiety, carrageenans are divided into families. Each structural disaccharide unit is then given a specific name based on the positions of the sulfate groups and whether or not the 3,6-anhydro sugar is present in the galactose moiety. The majority of carrageenans observed in nature have multiple carrabiose units, generating hybrid structures. The number of diads and their structure differ depending on the kind of algal species and their stage of life [90]. Carrageenans and agars have similar physical and chemical features that make them useful as gelling and stabilizing agents. Carrageenans have a high concentration of λ-, κ-, or ι-diad contents. Additionally, carrageenans and agars display a broad range of biological activities that are relevant to human health [91].

Carrageenan, a negatively charged, sulfated polysaccharide derived from red seaweed, is bioactive, abundant in nature, and has good biocompatibility. The isolated carrageenan from *Kappaphycus* has special anti-obesity potential, with the potential for multiple mechanisms of action, including the potential for discrete impacts on particular genes involved in lipid metabolism [92]. The red seaweed *Kappaphycus alvarezii* is the principal source of carrageenan [93]. Kappa (k)-carrageenan prepared from the red alga *Hypnea musciformis* was found to have antioxidant, antibacterial, anticancer, and neuroprotective properties [94]. Similarly, κ/β-carrageenan extracted from *Tichocarpus crinitus* showed immunomodulating properties [95]. The primary method for producing carrageenan oligosaccharides is the hydrolysis of their constituent polysaccharides [96]. Each repeating disaccharide unit in kappa-, iota-, and lambda-carrageenan has one, two, or three ester-sulfate groups, respectively. 

Their structural makeup was described as alternating 3-linked -D-galactose 4-sulfate and 4-linked AnGal units [97]. Two additional sulfates are present in each repeating unit of the disaccharide in the ι-carrageenans due to an extra sulfate group on C2(O) of the AnGal residue. ι-carrageenans isolated from *Eucheuma spinosum* were found to be more homogenous and flexible than κ-carrageenans based on an atomic force microscopy investigation [98]. There are no 3,6-anhydride bridges on the 4-linked residues of the λ-carrageenans, which have three sulfate groups per disaccharide unit, with the third sulfate group of this type at the C6 position. *Gigartina* and *Chondrus* species are used to make lambda-carrageenan [99]. The algae’s origins, the seaweed’s life-cycle stage, and the extraction procedures are all related to the heterogeneous chemical structures of carrageenan [100].

#### Sulfated Heteroglycans-Ulvans

**Figure 4:**
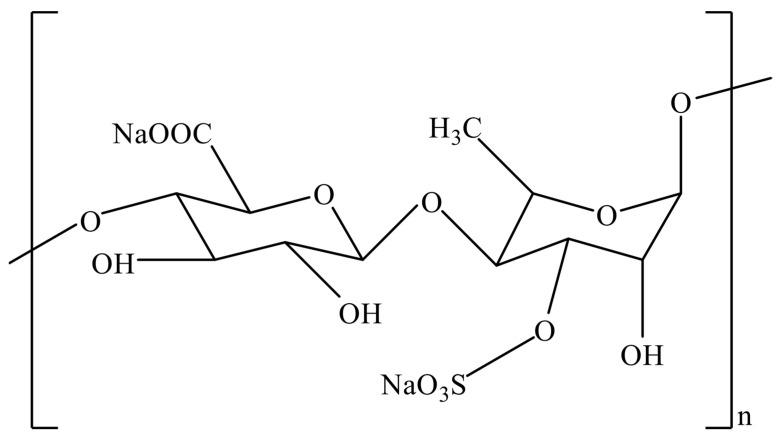
Ulvan primary structure.

Ulvan is a polysaccharide found in cell walls that makes up about 9 to 36% of the dry weight of Ulva’s biomass. It is mostly made up of uronic acids (glucuronic acid and iduronic acid), sulfated rhamnose, and xylose [101]. Rhamnose, xylose, glucuronic acid, and iduronic acid are the primary building blocks of the sulfated disaccharides that make up Ulvan. Both β-d-glucuronic acid (1 → 4)-α l-rhamnose-3-sulfate and α--l-iduronic acid (1 → 4)-α-l-rhamnose-3-sulfate are the primary repeating components. Instead of iduronic or glucuronic acid, the component may be a xylose unit (sulfated or non-sulfated), which produces the distinctive monomers β-d-xylose (1 → 4)-α-l-l-rhamnose-3-sulfate and β-d-xylose-2-sulfate(1→4)—l-rhamnose-3-sulfate (Figure 4). Ulvan contains minute quantities of galactose and glucose [102].

Advanced extraction and purification techniques, biomass species and quality, post-harvesting time, and pre-treatments can all have a substantial impact on the quantitative yield and quality of Ulvan [103,104]. 

#### 2.2.2. Seaweed Proteins and Peptides

In a cell, proteins are among the most prevalent macromolecules and are essential for both cell growth and the regulation of numerous metabolic processes. The global protein demand has increased, and seaweed-based protein has come under consideration as a substitute to meet the demand [105]. Proteins from seaweeds have been the focus of numerous studies due to their potential bioactivity. Overall, seaweeds contain 3–50% proteins in their dry weights, which is comparable with legumes and grains having high protein content [1,106]. However, the protein content varies from phylum to phylum, even within members of the same genera. Red seaweeds are reported to have a higher protein content (5–50%) compared with green (7–33%) and brown (3–22%) seaweeds. Moreover, protein contents vary in the same species if grown in different geographical locations [107]. Seaweed proteins can be classified into four parts: pigments, glycoproteins/lectins, enzymes, and peptides/amino acids [108]. Glycoproteins (GPs) are present on the algal cell surface and in the cell wall; they are sometimes secreted by tissues and can be obtained by water extraction. GPs are bound with carbohydrates through peptide chains by N-glycosyl linkages and/or O-glycosyl linkages. The presence of exclusively high-mannose glycans was revealed by the structural investigation of N-glycans in red, green, and brown seaweeds, and also N-glycans from zoostera marina and *Sargassum fulvellum* GPs found to have an M9 structure [109]. Seaweeds are reported as a source of novel lectins that make soluble and membrane-bound glycoconjugates through interaction with specific glycan structures [110]. As an illustration, griffithsin was discovered in the red algae *Griffithsia* spp.; HRL40 was discovered in *Halimeda renschii*; and SfL1 and SfL2 were discovered in *Solieria filiformis* [111,112,113]. Enzymes such as alkaline phosphatase, oxidases, and fibrinolytic enzymes are abundant in seaweeds. In green seaweed, Ulva species reported to have alkaline phosphatase and oxidases were found in *Caulerpa cylindracea*. In addition, *Codium fragile* and *Codium latum* are also known to have fibrinolytic enzymes (trypsin-like serine protease). Further, Rubisco has been isolated from the red seaweed *Kappaphycus alvarezii*. The amino acid composition of several species of macroalgae has been studied. In addition to being a rich source of acidic amino acids like aspartic acid and glutamic acid, seaweeds also contain significant levels of essential amino acids [114]. According to numerous studies, seaweeds contain between 41 and 50% essential amino acids of the total amino acids [115,116,117]. Aspartic and glutamic acids often make up about 25 to 40% of total amino acids in seaweed proteins [116,117]. These non-essential amino acids have intriguing flavor-development properties, and glutamic acid is a key element in the ‘Umami’ taste sense [33]. Among the seaweeds proteins, lectins and peptides are known to have several bioactivities (described in the biological activity of seaweeds section).

#### 2.2.3. Seaweed Pigments

Natural pigments occur in a wide range of colors and have been widely used in daily life throughout history. The manufacturing of food, the textile and paper industries, water science and technology, and agricultural research and practice are only a few areas worthy of consideration [118]. Pigments have advantageous biological activities as antioxidants and anticancer agents, in addition to having a number of critical qualities that make them suitable for use in these many industrial contexts. They thus have a tremendous potential to fulfill current economic needs, which have been progressively focusing on the health and biotechnology sectors in search of natural chemicals and products with established beneficial impacts on human health [118]. Seaweeds produce various kinds of pigments. Chlorophyll, phycobiliproteins, and carotenoids, including fucoxanthin, violaxanthin, antheraxanthin, zeaxanthin, lutein, and neoxanthin, are among the pigments found in seaweed. The phycobiliproteins are classified into three major classes, phycoerythrin (PE), phycocyanin (PC), and allophycocyanin (APC), representing one of the most important groups of seaweed proteins. They belong to the bilin family of fluorescent proteins, which are covalently joined to tetrapyrrole groups. In contrast to carotenoids and chlorophyll, which are found in the lipid bilayer, they make up a structure called phycobilisomes, that is linked to the cytoplasmic side of thylakoid membranes. Among the three classes, phycoerythrin (PE) is the most common and dominant protein in red seaweeds [5]. These pigments have drawn interest from the food and beverage sectors as well as from the animal feed, cosmetics, and pharmaceutical industries [119].

Fucoxanthin, the primary pigment that determines the color of brown macroalgae, is well known for being found in these organisms. Fucoxanthin covers the existence of other pigments from a macroscopic perspective and masks chlorophylls, regardless of the fact that the chlorophyll concentration of brown macroalgal species is usually greater. Chl a, Chl c, and β-carotene are all present in brown macroalgae in addition to fucoxanthin [120]. Though it is generally known that α- and β-carotenes, as well as their dihydroxy derivatives zeaxanthin and lutein, are the primary carotenoids in red macroalgae, several writers have stated that red algae has a more complex carotenoid profile, including fucoxanthin and neoxanthin, while others claim that zeaxanthin and lutein are indeed predominant [121]. Green macroalgae are similar to higher plants in their pigment composition, including lutein, violaxanthin, antheraxanthin, zeaxanthin, and neoxanthin. In contrast to terrestrial plants, green seaweed, such as *C*. *lentillifera*, has more xanthophylls, like canthaxanthin and astaxanthin [122]. Studies on green macroalgae have shown that the primary pigments responsible for the green color of these algae are chlorophyll and its derivatives [123]. The fucoxanthin extracted from the five brown seaweeds *Sargassum wightii*, *Sargassum ilicifolium*, *Sargassum longifolium*, *Padina* sp., and *Turbinaria* sp. showed antioxidant activity [124]. In previous studies, it has been proven that seaweed pigments show higher antimicrobial activity against Gram-positive bacteria (*Streptococcus agalactiae*, *S*. *epidermidis*, and *S*. *aureus*) compared to Gram-negative bacteria (*Escherichia coli*, *Klebsiella oxytoca*, and *K*. *pneumonia*) [125].

#### 2.2.4. Seaweed Lipid and Fatty Acid

Lipids are essential nutrients for humans. Fatty acids (FAs), which make up their constituents, can be categorized as saturated (SFAs; no double bonds), monosaturated (MUFAs; one double bond), and polyunsaturated (PUFAs; two to six double bonds). Humans are able to synthesize both SFAs and MUFAs. Despite this, since the human body is unable to synthesize PUFAs with the initial bond on the third or sixth carbon atom, they remain crucial. As a result, they must be obtained from food [70]. Seaweeds are also a sustainable alternative raw material and, in particular, a possible supply of polyunsaturated fatty acids (PUFAs) and healthy fats that may be utilized in a variety of high-end applications [126]. For functional food, cosmeceutical, nutraceutical, and feed enrichment uses, macroalgae’s lipids and biomass have been used increasingly often as ingredients [33].

The quantity of lipids in seaweed differs depending on the species, geographic area, season, salinity, temperature, and intensity of light, as well as the way these elements interact [127]. There are only a few lipids (1–8% of the dry weight of the macroalgae) present in macroalgae. However, the majority of them include PUFAs, including docosahexaenoic acid (C22:6n-3, DHA), eicosapentaenoic acid (C20:5n-3, EPA), and arachidonic acid (C20:4n-6, AA), all of which are extremely valuable as far as human and animal nutrition is concerned [128,129,130,131,132]. Essential fatty acids (EFAs) are now regarded as functional foods and nutraceuticals with a variety of health advantages, including the ability to lower the risk of diabetes, cancer, osteoporosis, and cardiovascular diseases (CVDs). In most Western nations, cardiovascular diseases are thought to be the primary cause of mortality [132].

The polyunsaturated 20-carbon fatty acids eicosapentaenoic acid (EPA, ω-3, C20:5) and arachidonic acid (AA, ω-6, C20:4) are the two that are most present in red seaweed. The predominant saturated fatty acid is palmitic acid (C16:0), which accounts for 26% of total fats, whereas oleic acid is a monounsaturated fat. Assays performed on *Neopyropia*/*Porphyra*/*Pyropia* sp. revealed that the main fatty acids were palmitic, eicosapentaenoic, arachidonic, oleic, linoleic, and linolenic acid [133]. Brown seaweed raised in temperate or subarctic regions is known to accumulate omega-3 and omega-6 PUFAs. Eicosapentaenoic acid (EPA, 20:5n3), stearidonic acid (SDA, 18:4n3), and a-linolenic acid (LNA, 18:3n3) are the three primary omega-3 PUFAs, whereas arachidonic acid (ARA, 20:4n6) is the main omega-6 PUFA [127,134]. Green algae are abundant in C18 polyunsaturated fatty acids, particularly α-linolenic (C18:3 n-3), stearidonic (C18:4 n-3), and linoleic (C18:2 n-6 acids) [135].

In a previous report, it was demonstrated that polyunsaturated fatty acids of *C*. *humilis* comprised 47.67% of total FAs, with arachidonic acid C20:4 (n-6) being the most abundant PUFA (18.1%) and eicosapentaenoic acid C20:5 (n-3) being the second-most prevalent PUFA (11.79%). In addition to having a high unsaturation index (UI = 191.42) and a low atherogenicity and thrombogenicity index (AI = 0.55 and TI = 0.04), *C*. *humilis* also had a low ω-6/ω-3 ratio and had promising antioxidant activity [136]. According to gas chromatography analysis, the most common monounsaturated fatty acid of *G*. *longissima* was oleic acid methyl ester (18:1), at 8.5%, whereas the most common saturated fatty acid was palmitic acid methyl ester (16:0). The fatty acid composition of *G*. *longissima* also showed an intriguing composition in polyunsaturated fatty acids; in particular, the ratio of ω-3 to ω-6 fatty acids was >1, suggesting that this macroalga may be exploited as a natural source of ω3 and as an antimicrobial agent due to its antimicrobial activity [137]. The fatty acid composition of *C*. *rupestris* lipidic extract includes linoleic, palmitoleic, palmitic, myristic, oleic, and α-linolenic acids. According to this study, *C*. *rupestris* lipidic extract is a viable source for upcoming biotechnological applications because of its antibacterial action, nutritional value, and bioplastic content [138]. The seaweed-sourced fatty acids are reported to have multiple bioactivities, such as antimicrobial, antioxidant, antifungal, antiproliferative, anti-parasitic, and anticancer activities (described in the biological activity of seaweeds section).

## 3. Secondary Metabolic Compounds in Seaweeds

Secondary metabolites are derived from primary metabolites and play crucial roles in several biological processes that help seaweeds adapt to their harsh environment. Secondary metabolites are classified into three main groups: alkaloids and glucosinolates, phenolic compounds, and terpenes. Secondary metabolites are crucial in various defense-related processes and primarily scavenge ROS to protect cells from lipid peroxidation [139]. Seaweeds are thought to be a significant source of natural antioxidants, as flavonoid and phenolic chemicals are two of the main contributors to antioxidant activity [140].

### 3.1. Phenolic Compounds

Phenolic compounds are metabolites with hydroxylated aromatic rings, where the hydroxyl group is directly linked to the phenyl, substituted phenyl, or other aryl groups [141]. Polyphenolic compounds are structurally diverse and include the flavonoids, phenolics, and terpenoids found in brown seaweed [142]. Phenolics are recognized antioxidant metabolites and include flavonoids, tannins, chalcones, and coumarins. Various types of flavonoids have been identified, such as anthocyanidins, isoflavones, flavanones, flavones, and flavonols [143]. Due to their numerous health benefits and intriguing bioactivity for prospective pharmaceutical applications, phenolics have received a lot of interest recently. The most researched seaweed polyphenols, phlorotannins, serve important ecological roles such as wound healing, herbivore protection, cell wall hardening, and hazardous metal chelating [144,145]. Phenolic compounds are found in low amounts in the genus *Gracilaria*. Bromophenols and benzoic acids are two of the few phenolics already identified in *Gracilaria* [146]. Monobromophenols such as 2-bromophenol and 4-bromophenol, and dibromophenols such as 2,4-bromophenol, 2,6-dibromophenol, and 2,4,6-tribromophenol, have been found in the genus *Gracilaria* [147]. In the genus *Gracilaria*, a few of the phenolic metabolites have been identified, such as salicylic acid, benzoic acid, vanillic acid, gallic acid, p-hydroxybenzoic acid, gentisic acid, protocatechuic acid, and syringic acid [148,149,150]. Key metabolites phlorotannins have been identified in brown seaweeds, which may have potential pharmacological activity [151,152,153]. Polyphenolics provide several potential uses in the nutraceutical, agricultural, pharmaceutical, and cosmetic industries.

Seaweeds produce polyphenols to shield themselves from herbivores and other biotic and abiotic stresses [154]. Phenolic compounds are SMs, mainly found in green and brown seaweeds [155]. The brown seaweed *Eisenia bicyclis* (Kjellman) Setchell had the highest polyphenol content, including gallate, epicatechin gallate, epigallocatechin, catechin, hesperidin, epicatechin, epigallocatechin, and morin, while the green seaweed *Caulerpa sertularioides* (Gmelin) Howe had tgallocatechin, gallocatechin gallate, and epicatechin [156]. Phlorotannins have been identified from the brown seaweed *Cystoseira trinodis* (Forsskal) Agardh and other Fucales species and have high antioxidant activity [157,158]. Phlorotannins extracted from *Eisenia bicyclis* (Ochrophyta, Phaeophyceae) have shown higher antioxidant activity than tocopherol and ascorbic acid [159]. Similarly, the flavone apigenin, isolated from *Acanthophora spicifera*, possesses anti-inflammatory and antinociceptive activities [160]. Seven polyphenols (myricetin, quercetin, caffeic acid, chlorogenic acid, phloroglucinol, gallic acid, and ferulic acid) were shown to have stronger antioxidant activity than ascorbic acid in the brown seaweed *Himanthalia elongata* [161]. Furthermore, polyphenols have a role in lipid peroxidation stabilization.

### 3.2. Terpenes

Terpenes are non-polar secondary metabolites that comprise isoprene units. They are hydrocarbons, which are the largest class of secondary metabolites [162]. Based on the isoprene units, terpenoids are classified into eight classes and have a wide range of bioactivities with pharmacological and nutraceutical benefits. The methylerythritol phosphate pathway produces monoterpenes (C10), diterpenes (C20), and triterpenes (C30), whereas the mevalonate pathway produces sterols, sesquiterpenes (C15), and triterpenes. Seaweed terpenes are a diverse and well-studied class of natural marine compounds with structures distinct from those of their terrestrial plant biosynthetic equivalents [163]. Brown seaweeds are thought to be one of the greatest sources of physiologically and ecologically significant terpenoids among macroalgae.

Monoterpenes are mainly reported from red seaweeds. The assembly of three isoprenoid units forms sesquiterpenes. Sesquiterpenes isolated from seaweeds may be classified into many groups based on their carbon skeletons, which include cuparane, laurene, chamigrane, and other skeletons [164]. *Plocamium*, *Portieria*, and *Ochtodes* are the only red macroalgae genera containing halogenated monoterpenes, a broad family of organohalogenated marine natural metabolites. Refs. [165,166] reported the presence of halogenated monoterpenes myrcene (one cyclic and three acyclic) in the seaweed *Ochtodes secundiramea*. Sesquiterpene elatol is a significant secondary metabolite identified in the red seaweed *Laurencia dendroidea* that protects against herbivory and fouling [167]. The sesquiterpenoid caulerpenyne is found in the green algae *Caulerpa racemosa* and *C. taxifolia* [168].

## 4. Biological Activity of Seaweeds

Seaweeds are very rich in their composition and have hugely diverse components. The seaweed composition varies not only with the group but also between species. Thus, different seaweed extracts, such as polysaccharides (Table 2), proteins and peptides (Table 3), and lipids and fatty acids (Table 4), show numerous bioactivities, such as antioxidants, antifungals, antivirals, anticoagulants, anticancer agents, and neuroprotective agents. Several reports have been published that highlight the biological activities of seaweed extracts (Table 2).

### 4.1. Antioxidant Activities

Reactive oxygen species (ROS) include free radicals such as hydroxyl radicals (OH^.^), anion radicals (O_2_^−^), and also various non-free radicals such as hydrogen peroxide H_2_O_2_, superoxide radicals (O_2_^−^), and singlet oxygen (^1^O_2_), which are generated in living organisms during metabolism and cause oxidative damage [240,241]. Oxidative stress induced by excessive ROS generation impacts biomolecules such as proteins, lipids, and nucleic acids and results in several chronic diseases, like cancer, cardiovascular diseases, atherosclerosis, neurological disorders, diabetes, muscular dystrophy, and aging [242,243]. Antioxidants are enzymatic and non-enzymatic molecules that protect the organism by inhibiting the harmful impacts of oxidative damage [244,245]. 

Antioxidants are frequently employed as food preservatives because they lower ROS and can stop food from spoiling. Synthetic antioxidants like tertiary butylhydroquinone (TBHQ), butylated hydroxyanisole (BHA), and butylated hydroxytoluene (BHT), which are all available for sale, are used as preservatives to keep food from going bad [246]. Seaweeds grow in intertidal to subtidal deeper zones and thus are exposed to different types of stresses, mainly desiccation, salinity, and temperature stress, resulting in higher production and accumulation of ROS. In order to cope with stresses, seaweeds synthesize antioxidant enzymes such as ascorbate peroxidase and catalase, as well as other compounds like pigments, polyphenols, polysaccharides, flavonoids, dietary fibers, minerals, and essential amino acids, which are non-enzyme antioxidants [245,247]. Several reports have been published highlighting the antioxidant potential of red, green, and brown seaweed extracts using in vitro assays like 2,2-diphenyl-1-picrylhydrazyl (DPPH), the ferric-reducing ability of plasma (FRAP), and 2,2′-azino-bis (3-ethylbenzothiazoline-6-sulfonic acid) (ABTS) radical-scavenging activity [248]. Various polysaccharides extracted from red seaweeds, such as agar [249], carrageenan [250], and porpohyran [251], and oligosaccharides like agar-oligosaccharide [252] and carrageenan oligosaccharide [253] were reported to have antioxidant capacity, determined using in vitro assays. In brown seaweeds, alginate and their oligosaccharides [254,255], fucoidan and fucoidan oligosaccharides [85,256], and laminarin and laminarin oligosaccharide [257,258] were found to have high antioxidant capacity. Ulvan, a green seaweed polysaccharide, and the oligosaccharide prepared from it tested positive for antioxidant capacity. Other than carbohydrates, seaweed proteins, lipids, phenolic compounds, and pigments, such as chlorophyll, phycobiliproteins, carotenoid, and fucoxanthin, are also known to have antioxidant potential [245]. For example, glycoproteins from two brown seaweeds, *Fucus serratus* and *Fucus vesiculosus*, and peptides from *Pyropia columbina* have been reported to have good antioxidant properties [220,222] (Table 3). Also, omega-3 PUFA from brown seaweed *Undaria pinnatifida*, palmitic acid from green seaweeds *Caulerpa racemosa*, *S. tenerrimum*, *C. indica*, *C. veravalnensis,* and levoglucosenone, and 4-pyridinemethanol and hexamethyl-cyclotrisiloxane from *K*. *alvarezii* are known to have antioxidant properties (Table 4). A few in vivo studies also showed the antioxidant potential of various seaweed extracts [85,259,260,261,262,263]. Both in vitro and in vivo studies have reported on different seaweed extracts, indicating their potential as antioxidants for human health. However, techniques still need to be improved to purify the selected components and commercialize them for health benefits.

### 4.2. Anticoagulant Activity

Anticoagulants are compounds that work as blood thinners and help prevent blood clots. Sulfated polysaccharides are widely studied for their anticoagulant activities. Heparin is a sulfated polysaccharide currently used as an anticoagulant drug in most clinical practices. Heparin is a member of the glycosaminoglycan family and is composed of d-glucosamine-N-sulfate, 6-O-sulfate, and l-iduronic-2-O-sulfate acid [264]. The side effects, such as hemorrhage and thrombocytopenia from heparin, draw attention to the search for a safe alternative [265]. All three groups of seaweeds are known to contain sulfated polysaccharides; for example, green seaweeds are the source of ulvan, red seaweeds contain carrageenan, agar, and porphyran, and brown seaweeds are known sources of fucans/fucoidans [266]. These polysaccharides have gained huge attention among researchers and have been investigated to develop alternative drugs for heparin with no or fewer side effects [52]. Chemical assays such as activated partial thromboplastin time (APTT), thrombin time (TT), and prothrombin time (PT) are used to analyze the anticoagulant potential of compounds. Among green seaweed, sulfated polysaccharide ulvan from *Ulva fasciata* was reported to have a prolonged APTT of 88–92 s [160]. Similarly, polycarboxyl ulvans prepared with the same species showed clotting times in the range of 101–227 s, which was 7.7 to 17.9 times higher than native ulvans [267]. In another study, 75 µg/mL of polysaccharide extracted from *Monostroma nitidum* showed 107%, 120–123%, and 167% higher inhibition activities for APPT, PT, and TT assays, respectively, compared to standard heparin [268]. Polysaccharide from *Monostroma angicava* was also found to be effective in increasing the clotting time in APPT and TT assays and exhibited good anticoagulant activity in in vitro and in vivo studies [269]. Further polysaccharides from the red seaweed *Agardhiella subulata* showed a prolonged clotting time >100 s in APPT assay [199], and carrageenans extracted from different species, such as *Hypnea valentiae* (Turnur) [193], *Kappaphycus alvarezzi*, *Gigartina skottsbergii* (tetrasporic phase), and *Eucheuma denticulatum,* as well as carrageenan derivatives, were found to have good anticoagulant activity [270]. Further, a 50 μg μL^−1^ concentration of fucans extracted from brown seaweed *Lobophora variegata* showed an increase in clotting time of 250 s related to the control, suggesting the optimum concentration led to the maximum increase in clotting time [270]. Also, peptides from the red seaweed *Porphyra yezoensis* were found to have anticoagulant properties [217]. Overall, the research points to the possibility that sulfated polysaccharides could delay the APTT, PT, and TT stages of the coagulation cascade and prevent blood coagulation.

### 4.3. Anticancer Activity

Components of all three types of seaweed—green, red, and brown—are investigated for their potential to act as an anticancer agent. Seaweeds are made up of several components; minor components are chlorophylls, carotenoids, phycobiliproteins, phlorotannin, terpenes, polyphenols, and lipids, while major components are polysaccharides, proteins, and minerals. Among these components, polysaccharides such as sulfated galactans from red seaweeds, fucoidan and alginic acid from brown seaweeds, and ulvan extracted from green seaweeds are studied for their anticancer activity. Other than polysaccharides, minor components of seaweed biomass, such as terpenes, polyphenols, and fucoxanthins, were also reported to have anticancer activity. In green seaweeds, Ulvan polysaccharide extracted from *Ulva lactuca* showed in vitro and in vivo activity with chemopreventive efficiency against breast carcinogenesis [271,272]. The research also concluded that the antioxidant defense system suppressed oxidative stress, augmented apoptosis, and reduced inflammation. Among the red seaweeds, carrageenans extracted from *Kappaphycus alvarezii* were reported for their inhibitory effect on the growth of breast, colon, liver, and osteosarcoma cell lines [189,273]. Sulfated galactan extracted from *Gracilariopsis lemaneiformis* inhibited the viability of A549, B16, and MKN-28 cell lines [274]. Further sulfated polysaccharides obtained from *Amansia multifida* inhibited the viability of HeLa cells and decreased the viability of mouse 3T3 fibroblasts, an undesirable effect of an anticancer drug [275]. Similarly, low-molecular-weight polysaccharides prepared from the seaweed *Gayralia oxysperma* were found to inhibit U87MG glioblastoma cell viability without affecting normal Vero cells [276]. In brown seaweeds, Vishchuk et al., 2011 [277], extracted fucoidan from *Undaria pinnatifida* and treated the T-47 and SK-MEL-28 cancer cell lines, resulting in 46% T-47D and 34% inhibition of SK-MEL-28. However, fucoidans inhibited colony formation by 79% and 51% for the tested cell lines, respectively. In another experiment, DLD-1, T-47D, and RPMI-7951 cell lines were treated with fucoidan, and treatment inhibited the growth of RPMI-7951 and T-47D by 40 and 60%; however, no inhibition was observed in DLD-1 cell lines [278]. Apart from these glycoproteins from *Codium decorticatum* [214] and *Laminaria japonica* [225], lectin from *Solieria filiformis* [112] peptides from *Porphyra yezoensis* [223] was also found to have anticancer activities. Further, 3(ζ)-hydroxy-octadeca-4(E),6(Z),15(Z)-trienoic acid (1) and 3(ζ)-hydroxy-hexadeca-4(E),6(Z)-dienoic acid esters from *Tydemania expeditionis* were reported to have antitumor activity [239]. Seaweed extracts work as anticancer agents, as they enhance immune functions, inhibit cancer cell invasion and metastasis, substantially induce apoptosis of cancer cells, and also scavenge free radicals [279]. To date, several studies have highlighted the anticancer activities of seaweeds; however, these leads are still awaiting confirmation as cancer drugs. 

### 4.4. Neuroprotective Activity

Neurodegenerative diseases such as Huntington’s disease, Parkinson’s disease, Alzheimer’s disease (AD), and amyotrophic lateral sclerosis (ALS) are widespread diseases, wherein certain neurons are irreversibly damaged, resulting in the loss of nervous system functions [280,281]. Several synthetic and natural neuroprotective agents are used to avoid or treat such diseases. However, synthetic agents are considered to cause side effects like nervousness or anxiety, drowsiness, redness, and balance difficulties. In order to overcome these effects, attention turns toward natural and safe agents. Alcoholic extracts from different species of red, green, and brown seaweeds have been studied using in vitro assays or animal models for their potential as neuroprotective agents [282,283,284,285,286,287]. Apart from these, seaweed-based sulfated polysaccharides, such as fucoidans and alginic acid from brown seaweeds [288,289], kappa, lambda, and iota-carrageenan from red seaweeds [45], and ulvan from green seaweed [218] also showed neuroprotective activities. Seaweed extracts and polysaccharides prevent excessive ROS generation, thus protecting the body from oxidative damage and neuro-inflammation [290]. One of the most common carotenoids found in brown seaweeds, fucoxanthin, has been shown to have neuroprotective activity that crosses the blood–brain barrier and exerts anti-neurodegenerative disease effects more effectively than the other carotenoids [291]. It’s effects impact such elements as oxidative stress, amyloid protein aggregation, neuroinflammation, dysregulation of neurotransmission, neuronal death, and dysbiosis of the gut. The research on several seaweed extracts and polysaccharides has shown positive effects; these compounds could be an effective treatment for neurodegenerative diseases. However, most of the investigations focused on in vitro assays and animal models. Thus, adequate clinical trials could help the development of effective neuroprotective drugs.

### 4.5. Antiviral Activity

Like several other biological activities, seaweed extracts from green, red, and brown seaweeds are also reported to have antiviral activities [292]. During the early 1990s, sulfated polysaccharides prepared from two seaweed species, *Aghardhiella tenera* and *Nothogenia fastigiate,* were found to have antiviral activity against respiratory syncytial virus (RSV), herpes simplex virus (HSV-1 and HSV-2), and human immunodeficiency virus (HIV), which are human infectious viruses [293,294,295]. Sulfated polysaccharides from seaweeds were also found to be effective against dengue virus (DENV)-2, which prevents the adsorption and internalization of the virus [296]. In recent years, several published articles have also highlighted the antiviral activity of seaweed extracts against different viruses [297]. For example, Gomaa and Elshoubaky, 2016 [298], extracted sulfated polysaccharides from two seaweed species, *Acanthophora specifira* (red) and *Hydroclathrus clathratus* (brown alga), which were found to be effective in inhibiting the human pathogenic virus HSV-1 and Rift valley fever virus (RVFV). Iota-carrageenan and its N-sulfonated derivatives of poly (allylamine) hydrochloride were found to have antiviral properties against the human metapneumovirus (hMPV) [299]. Several human and avian influenza viruses have been found to be resistant to the effects of ulvans isolated from *U*. *lactuca* and *U. clathrata* grown in labs, as well as a combination of ulvans and fucoidans isolated from *Cladosiphon okamuranus* [300,301]. Iota-carrageenan and its derivatives blocked virus release from the cellular membrane and also inhibited virus adsorption. Further, Cirne-Santos et al., 2019 [302], prepared an ethanolic extract from the red seaweed *Osmundaria obtusiloba* (C. Agardh) and tested the inhibitory effects against the chikungunya virus (CHIKV). A high level of inhibition was demonstrated by *O*. *obtusiloba* extract, with 420 as the selectivity index and 1.25 g mL^−1^ as the inhibition value. More recently, Chen et al., 2020 [303], reported the antiviral activity of sulfated polysaccharides against coronavirus disease 2019 (COVID-19). These sulfated polysaccharides are known to obstruct the entry of viruses into cells [304] and also inhibit viral cell autophagy [305]. Further fucoidans and phlorotannins from brown seaweeds and carrageenan were also found to be active against SARS-CoV-2 [306,307]. The recent investigation of the antiviral activity of seaweed and seaweed compounds suggests their potential application as antiviral drugs against COVID-19 in the near future [308].

### 4.6. Antifungal Activity

Aqueous and organic solvent extracts from seaweeds are widely investigated for their antifungal activities. Ambika and Sujatjha, 2015 [309], prepared aqueous and alcoholic extracts from two seaweeds, *Sargassum myriocystum*, and *Gracilaria edulis,* and tested them against the sugarcane pathogen *Colletotrichum falcatum* mycelial growth. The ethanol extracts from *S*. *myriocystum* effectively inhibited mycelial growth. Lotfi et al., 2021 [310], extracted metabolites using organic solvents from three seaweed species, *Cladophora sericea*, *Ulva lactuca*, and *Ulva fasciata,* and investigated the antifungal activity against *Macrophomina phaseolina* (Tassi) Goid. and *Fusarium oxysporum* Schltdl. The *U*. *fasciata* acetone extract was found to have the highest activity against both of the tested fungi. Recently, methanol and hexane extracts from *Amphiro aanceps* were tested against three fungi, *Fusarium nculmorum*, *Rhizoctonian solani*, and Botrytis cinerea. The maximum antifungal activity was found against *B*. *cinerea*, and the minimum was against *F*. *culmorum* [311]. Similarly, *Ulvafasciata* extract was found to have good biological activity, with a 69.26% inhibition rate against *B*. *cinerea* [311]. The fatty acids from several seaweeds, such as *Spatoglossum asperum*, *Ceramium rubrum*, *Ulva lactuca*, *Sargassum tenerrimum,* and *Laurencia obtuse,* were also found to have antifungal activities (Table 4). Overall, research on seaweed extracts has shown their potential as a biological control against various fungi.

### 4.7. Anti-Diabetic Properties

Diabetes is the most common disease occurring worldwide and is known to develop metabolic disorders in protein, carbohydrates, and fat [312]. It is characterized by a high level of blood sugar, called “hyperglycemia”, which occurs due to the reduced secretion of insulin from β-cells [313]. Generally, synthetic hypoglycemic drugs are used to manage diabetes; however, regular use of these drugs has several side effects. Thus, attention has been turned to finding natural alternatives with anti-diabetic potential [314]. Seaweeds, being a known source of various bioactive compounds, are also investigated for their potential as anti-diabetic agents. In several recent studies, crude extracts from green, red, and brown seaweeds [315,316,317,318] and also a few specific compounds, such as phlorotannins, fucoxanthin, and polysaccharides [319,320], were evaluated for anti-diabetic properties such as α amylase and α glucosidase inhibitory activities using in vitro assays and showed significant inhibition. Further in vivo studies were also carried out in animal models, which suggested that seaweed extracts not only ameliorate hyperglycemia, hyperlipidemia, and liver and kidney damage but are also effective in controlling free fatty acids (FFAs), aspartate aminotransferase (AST), alanine aminotransferase (ALT), uric acid (UA), and total bile acid (TBA) in diabetic rats [320,321,322,323]. Although the precise mechanism underlying the anti-diabetic activity of seaweed extracts and compounds is unknown, it is most likely a result of the antioxidant enzymes that scavenge free radicals and lessen hyperglycemia brought on by oxidative stress and the associated hyperlipidemia. Seaweed-based extracts and compounds also block carbohydrate-hydrolyzing enzymes in vitro and lower blood glucose levels in vivo in random and postprandial blood glucose testing. In certain animal studies, they have also been found to reduce weight gain, most likely via decreasing the mRNA expression of pro-inflammatory cytokines while increasing the mRNA expression of anti-inflammatory cytokines [314]. Recently, a seaweed-based antidiabetic medicine was developed and commercialized under the brand name Cadalmin^TM^ Antidiabetic Extract for use against type II diabetes (https://www.cmfri.org.in/cmfri-nutraceuticals, accessed on 23 August 2023). However, there is a need to conduct more extensive research in this field.

### 4.8. Anti-Obesity

Obesity has become an epidemic with global spread. It is a major threat to human health and is connected with type 2 diabetes, metabolic syndromes, and cardiovascular disease. Currently, obesity is managed with the use of a small number of available weight-loss drugs, such as orlistat, locaserin, phentermine/topiramate, and naltrexone/bupropion, along with exercise and a proper diet. Based on the demands for more new and safe anti-obesity drugs, brown seaweeds and seaweed-based compounds such as phlorotannins, fucoidans, fucoxanthin, and alginates were evaluated for their anti-obesity properties. The majority of studies are conducted in vitro, in vivo, and in animal models, either feeding whole seaweed meals or selective seaweed extracts or compounds [324]. For example, feeding *Undaria pinnatifida* to diet-induced obese mice positively affected body weight gain, energy consumption, and glucose and insulin serum levels [325]. In another study, feeding *Sargassum polycystum* powder to rats fed a high-fat diet suppressed weight gain and also reduced plasma levels of cholesterol and triacylglycerols [326]. Further studies conducted on humans showed a 16.4% reduction in energy intake among those given breakfast bread enriched with the brown seaweed *Ascophyllum nodosum* [327]. In seaweed-based extracts, ethanolic extract from the red seaweed *Grateloupia elliptica* was found to increase anti-adipogenic activity in 3T3-L1 cells and in mice with high-fat diet (HFD)-induced obesity by suppressing the expression of adipogenic proteins and inhibiting intracellular lipid accumulation [328]. The in vivo experiment showed that seaweed extract significantly reduced body weight and adipose tissue (WAT) and also lowered the levels of serum triglycerides, total cholesterol, and leptin contents. Similarly, a 40% ethanol extract from the red seaweed *Plocamium telfairiae* was found effective in alleviating lipid droplet accumulation in 3T3-L1 adipocytes and obese C57BL/6 mice [329]. The mechanisms of action of seaweed and seaweed-based compounds against obesity reported in various articles include the inhibition of pancreatic lipase, suppression of adipocyte differentiation, and inflammation in white adipose tissues (WAT) by downregulation of the responsible genes and enhanced ß-oxidation through increased expression of uncoupling protein 1 [324,328,329]. Several in vitro and in vivo studies showed the potential of seaweeds and their selective compounds as anti-obesity agents; however, there is a need to conduct extensive studies and clinical trials to confirm their usability as anti-obesity drugs.

### 4.9. Anti-Inflammatory Activities

A major contributor to global health problems and a significant driver of rising healthcare expenses is inflammatory disorders. Natural molecules are always advantageous when compared with synthetic ones, as they have fewer side effects when used in therapeutics. Marine-algae-based compounds such as polyphenols, sulfated polysaccharides, terpenes, fatty acids, and proteins are a source of unique anti-inflammatory properties [330]. The anti-inflammatory properties of seaweed lipids were found to downregulate the production of various pro-inflammatory cytokines, including TNF, IL-1, IL-6, IL-8, MCP-1, and NO. Additionally, the expression of several inflammatory cytokines, namely iNOS, COX-2, IL-6, IL-8, and MCP-1, was also downregulated [331]. Fernando et al., 2016 [330], found that sulfated polysaccharides sourced from *Lobophora variegata*, *Fucus vesiculosus*, *Porphyridium*, *Gelidium crinale*, and *Porphyra yezoensis* seaweeds were also isolated and found to have intra-inflammatory properties. Proteins, peptides, and amino acids are also reported to have anti-inflammatory properties. For example, lectin from the green seaweed *Caulerpa cupressoides* has been reported to inhibit paw oedema in mice [332]; lectin from the red seaweed *Pterocladiella capillacea* inhibited neutrophil migration [333]; and lectin sourced from *Hypnea cervicornis* was found to inhibit carrageenan and ovalbumin-induced hypernociception in rats [334]. Other than lectin, purified glycoproteins from *Porphyra yezoensis* are also reported to have anti-inflammatory activity [335]. Phenolic compounds such as phlorofucofuroeckol A and B, phloroglucinol, vidalols A and B, eckol, dieckol, 7-phloroeckol, dioxinodehydroeckol, diphlorethohydroxycarmalol, octaphlorethol A, catechol, and rutin from seaweeds were found to have anti-inflammatory properties [336,337,338,339,340]. Terpenoid compounds from seaweeds are considered effective anti-inflammatory agents because it has been demonstrated that several of them play a significant function as modulators in the NF-kB signaling pathway [341]. The known mode of action of algal compounds is the suppression of pro-inflammatory cytokines. Their potential utility as a novel functional food ingredient and/or health supplement, as well as the intricate mechanisms behind their preventive activity against chronic inflammation, need more study.

## 5. Challenges

### 5.1. Efficient Green Methods for Compound Extraction and Purification

Seaweed compounds are extracted using different methods and a variety of solvents. Hot water extraction and occasionally alkali or acid treatments are used to extract the majority of sulfated polysaccharides. Pigments are extracted using various extractives, either alone or in combination. Chlorophyl and fucoxanthins are extracted with organic solvents, while phycobilliprotein is extracted with water or buffers. The lipids are extracted using the solvent chloroform and methanol. The yields and bioactivity of seaweed compounds greatly varied with the extraction methods [342]. There are several biological activities reported with crude extracts instead of pure compounds. There is a need to develop compound-specific green extraction and purification methods in order to use these compounds in various applications, such as food, pharmaceuticals, nutraceuticals, and cosmetics [248,343].

### 5.2. Toxicity and Antinutrients

Today, seaweed is consumed both as a whole food and as a component in a wide variety of foods. Seaweeds are common in coastal locations, and numerous studies have shown the advantages they offer for human health [344]. With the increasing demand for seaweed for edible uses, concern is developing about the safety of its consumption. Wu et al., 2022 [345], discussed the food safety risks associated with algal consumption in detail and classified them into three classes: (1) physical factors, including radioactive and microplastic contaminants; (2) chemical hazards, including iodine, heavy metals, sulfur dioxide, pesticide residues, and veterinary drug residue; and (3) biological hazards, including pathogenic bacteria and algal toxins. There are few studies conducted in nuclear power plant–affected areas in Japan that identified the presence of radioactive contaminants with higher than permitted values in edible seaweeds [346,347]. Li et al., 2020 [348], investigated the presence of microplastics in the commercial seaweed Nori. Further, seaweeds are known to have a tendency to absorb heavy metals in high concentrations when grown in metal-polluted waters. The metal content is higher than the permissible limit, resulting in toxicity. The metals responsible for the toxicity are arsenic, mercury, cadmium, nickel, lead, chromium, and iodine, when present above the permissible limit. There have been several studies conducted to evaluate the toxicity of seaweed, either as a whole or as individual components [24]. For example, Ma et al., 2018 [349] reviewed about 282 species belonging to green, red, and brown seaweeds and found that 10 species of brown seaweed have an arsenic content of more than 100 mg kg^−1^ dry seaweed. Similarly, Filippini et al., 2021 [350], analyzed 72 seaweed samples of European and Asian seaweed. The seaweeds belonging to the Rhodophyta were found to contain high levels of hazardous metals and trace elements. Also, the study results emphasize the importance of monitoring dangerous metal concentrations in food, particularly in edible seaweeds, where high levels of Al, Cd, As, and I have been linked to health hazards. On the other hand, there is very limited research on anti-nutrients in seaweeds. One detailed study reported on anti-nutrients in drifted seaweed on the Ceará Coast in Brazil [351]. As per the study, anti-nutrient and/or toxic factors, such as trypsin and a-amylase inhibitors, polyphenol compounds (tannins), lectins, phytic acid, and toxic contaminants (heavy metals), are found in seaweed, which can lessen its nutritional quality [351]. There is a need to avoid the polluted areas when cultivating seaweeds. If seaweed is contaminated, toxins must be removed before it is consumed. Traditionally, boiling edible seaweed kombu in water for 15 to 30 min seems to reduce (remove up to 99%) the amount of iodine. Recently, Wang et al., 2022 [352], successfully removed 98% of arsenic from the edible seaweed *Sargassum fusiforme* by sequential processing. In the first step, *S*. *fusiforme* was treated with hot water, and in the second step, it was treated with citric acid, followed by fermentation using the microbe *Lactobacillus rhamnosus*. Further research is needed to evaluate the anti-nutrients in seaweeds. It is vital to address the problem of quick and accurate detection of harmful and pathogenic microbial contamination in algal products. The presence of heavy metals and anti-nutrients in seaweeds restricts their use for direct consumption and in crude form. Furthermore, efficient purification is needed before their application in nutraceuticals. The development of cutting-edge technologies for monitoring heavy metals, algal toxins, and other contaminants is urgently needed.

### 5.3. Need for Unified Global Regulations

According to the FAO report [353], there is no standard global regulation available for the use of seaweed and seaweed-based products in food applications. However, certain countries have adopted regulations for food safety hazards in seaweed. The European Union has granted CEN/TC 454 a mandate to work on standardizing algae and algal products, which includes creating standards for areas where seaweed analysis methods are lacking (such as species identification, pigments, sugars, proteins, and lipids). Recently, the FDA officially classified unprocessed seaweed as a raw agricultural commodity (RAC) in the United States. This certification mandates that unprocessed seaweed sold in the nation adhere to the general regulations of the Federal Food, Drug, and Cosmetic Act (FFD&C Act 402(a)). France has similarly implemented maximum restrictions for inorganic arsenic, cadmium, lead, and mercury in edible seaweeds. Cadmium has also been given a regulatory limit in China. The decrease in consumer-level food safety risks in seaweed has also been the subject of a number of consumption advisory notices, including in Japan, Ireland, and Norway. There is an urgent need to have standard global regulations for the use of seaweed as food.

## 6. Conclusions

The following may be deduced from a thorough examination of the publications on functional foods, biological activities, and nutritional potentials: 1. Seaweed has a high potential for nutraceuticals, and eating it directly has many positive health effects. 2. There is a need for technological inventions to extract and purify pure bioactive compounds for nutraceutical and pharmaceutical applications. 3. Several research articles highlighted seaweed components’ potential as anticancer, neuroprotective, anticoagulant, and antiviral agents; however, in vivo studies and adequate clinical trials are needed to translate the leads into drugs. Additional research is necessary to fully understand the impacts and underlying processes associated with seaweed. This will facilitate the utilization of biologically active compounds produced from marine sources, including micro- and macroalgae, in the advancement of therapeutic interventions and the production of functional food products.

## Figures and Tables

**Table 1:** Macro- and micromineral content in different seaweeds.

No.	Species	Key Minerals in Selected Seaweeds								Reference
		Ca (mg/ 100 g DW)	Mg (mg/ 100 g DW)	K (mg/ 100 g DW)	Na (mg/ 100 g DW)	Cu (mg/100 g DW)	Zn (mg/100 g DW)	Fe (mg/ 100 gDW)	I (mg/100 g DW)	
1	*Padina australis*	28.3	4	0.5	1	0.005	0.013	0.446	-	[26]
2	*Fucus vesiculosus*	938	994	4322	5469	<0.5	3.71	4.2	-	[26]
3	*Laminaria digitata*	1005	659	11,579	3818	<0.5	1.77	3.29	-	[27]
4	*Chondrus crispus*	420	732	3184	4270	<0.5	7.14	3.97	-	[27]
5	*Porphyra tenera*	390	565	3500	3627	<0.5	2.21	10.3	-	[27]
6	*Caulerpa racemosa*	-	-	-	-	5.35	2.96	404.94	-	[28]
7	*Ulva lactuca* CRM 279	-	-	-	-	13.83	76.88	883.28	-	[28]
8	*Colpomenia sinuosa*	5226.9	-	3510	423.83	0.94	-	1581	-	[29]
9	*Dictyota dichotoma*	3256.5	-	3417.01	633.85	1.29	-	1196	-	[29]
10	*Caulerpa lentillifera*	1874	1029	1143	8917	0.11	3.5	21.4	-	[30]
11	*Porphyra columbina*	443.7	491.53	1444.17	414.22	0.51	1.46	22.0	-	[31]
12 *	*Halimeda opuntia*	-	20.14	91.74	34.76	8.93	0.72	19.88	-	[32]
13 *	*Kappaphycus alvarezii*	-	90.5	672	25.66	5.25	7.75	8.15	-	[32]
14 **	*Ascophyllum nodosum*	575	225.0	765.0	1173.8	0.8	-	14.9	18.2	[33]
15 **	*Undaria pinnatifida*	112.3	78.7	62.4	448.7	0.2	0.3	3.9	3.9	[33]
16 **	*Chondrus crispus*	373.8	573.8	827.5	1572.5	0.1	-	6.6	6.1	[33]
17 ***	*Eucheuma cottonii*	329.69	271.33	13,155.2	1771.84	0.03	4.30	2.61	9.42	[34]
18 ***	*Caulerpa lentillifera*	1874.74	1028.6	1142.68	8917.46	0.11	3.51	21.37	4.78	[34]
19 ***	*Sargassum polycystum*	3792.06	487.81	8371.23	1362.13	0.03	2.15	68.21	7.66	[34]
20	*Ulva intestinalis*	794.5	4115.2	2456.8	1711.9	0.6	1.5	-	-	[35]

* Represents weight in mg/kg; ** represents weight in mg/100 g wet weight; *** represents weight in μg g^−1^ DW.

**Table 2:** Seaweed polysaccharides: sources and their biological activities.

Seaweed Species	Polysaccharide	Activities	References
*Undaria pinnatifida*, *Fucus vesiculosus*	Fucoidan	Anticancer activity against MCF-7 and ZR-75D breast cancer	[169]
*Sargassum polycystum*	Fucoidan	Antioxidant and Anticancer activities	[170]
*Scytosiphon lomentaria*	Fucoidan	Antiviral activity against HSV-1 and HSV-2	[171]
*Laminaria japonica*	Fucoidan	Anticoagulant and Antithrombotic activities	[172,173]
*Hizikia fusiforme*, *Laminaria japonica*	Fucoidan	Antioxidant activity	[85,174]
*Eclonia cava*, *Sargassum hornery*, *Costaria costata*, *Cladosiphon okamuranus*	Fucoidan	Antitumor activity	[175,176,177]
*Sargassum wightii*, *Undaria pinnatifida*	Fucoidan	Immunomodulatory activity	[178,179]
*Ecklonia cava*, *Lessonia vadosa*	Fucoidan	Anticoagulant activity	[180,181]
*Chondracanthus teedei var. lusitanicus*	Carrageenan	Antifungal activity	[182]
*Laurencia papillosa*, *Ascophyllum nodosum*, *Undaria pinnatifida*	Carrageenan	Antiproliferative activity	[183,184]
*Eucheuma Cottonii*	Carrageenan	Wound healing	[185]
*Lomentaria catenata*	Carrageenan	Anticoagulant activity	[186]
*Solieria filiformis*	Carrageenan	Herpes simplex virus type 1 (HSV-1) and Antioxidant activities	[187]
*Chondrus ocellatus*	Carrageenan	Antitumor and Immunomodulation activities	[188]
*Kappaphycus alvarezii*, *Portieria hornemannii*, *Spyridia hypnoides*, *Asparagopsis taxiformis*, *Centroceras clavulatum*, *Padina pavonica*	Carrageenan	Antioxidant, Anticancer, and Antidiabetic activities	[189,190,191]
*Hypnea valentiae*	Carrageenan	Antimicrobial, Antioxidant, and Anticoagulant activities	[192]
*Ulva lactuca*	Ulvan	Antioxidant properties, Cytotoxic activity Anti-proliferative and Apoptotic effects	[193,194,195]
*Ulva armoricana*	Ulvan	Antiviral and Antioxidant activities	[196]
*Ulva fasciata*	Ulvan	Anti-hypercholesterolemic and Antidiabetic activities	[197]
*Capsosiphon fulvescens*, *Ulva fasciata*, *Enteromorpha prolifera*	Ulvan	Anti-coagulant activity	[198,199,200]
*Ulva reticulate*, *Ulva armoricana*	Ulvan	Antimicrobial activity	[201,202]
*Ulvaintestinalis*, *Ulva pertusa*, *Ulva intestinalis*	Ulvan	Immunomodulatory activity	[203,204,205]
*Ulva pertusa*, *Enteromorpha linza*	Ulvan	Antioxidant activity	[206,207]
*Ulva pertusa*, *Enteromorpha prolifera*	Ulvan	Antioxidant and Antihyperlipidemic activities	[208,209,210,211]
*Enteromorpha intestinalis*, *Ulva fasciata*	Ulvan	Anticancer activities	[212,213]

**Table 3:** Seaweed proteins and peptides: sources and their biological activities.

Seaweeds	Proteins and Peptides	Biological Activity	References
*Codium decorticatum*	Glycoprotein	Anti-cancer activities	[214]
*Caulerpa cupressoides*	Lectin	Anti-nociceptive and anti-inflammatory	[215]
*Capsosiphon fulvescens*	Glycoprotein	Anti-aging agent	[216]
*Porphyra yezoensis*	Peptide	Anticoagulant activity	[217]
*Halimeda renschii*	Glycoprotein	Antiviral activity	[113]
*Saccharina longicruris*	Peptides	Antibacterial	[218]
*Solieria filiformis*	Lectin	Anticancer activity	[112]
*Cratylia floribunda*, *Vatairea macrocarpa*, *Bauhinia bauhinioides*, *Bryothamnion seaforthii & Hypnea musciformis*	Lectin	Antimicrobial activity	[219]
*Pyropia columbina*	Peptides	Antioxidant and ACE I inhibitory	[220]
*Undaria pinnatifida*	Glycoprotein	Anti-Alzheimer’s and anti-inflammatory activities	[221]
*Fucus serratus & Fucus vesiculosus*	Glycoprotein	Antioxidant activity	[222]
*Porphyra yezoensis*	Peptides	Anticancer activity	[223]
*Saccharina japonica*	Glycoprotein	Probiotic properties	[224]
*Laminaria japonica*	Glycoprotein	Anticancer activity	[225]
*Palmaria palmata*	Protein fraction	Cardioprotective, anti-diabetic, and antioxidant activity	[226]

**Table 4:** Fatty acids and lipids: sources and their biological activities.

Seaweed	Lipid/Fatty Acid	Biological Activity	References
*Gracilaria vermiculophylla*, *Porphyra dioica* and *Chondrus crispus*	Palmitic acid (16:0)-(PUFA); monounsaturated fatty acids (MUFAs)	Antimicrobial activity	[227]
*Ulva linza*, *Sargassum vulgar,* and *Gracilaria corticata*	C18:1n-9- MUFA and C18:4-PUFA	Antibacterial activity	[228]
*Undaria pinnatifida*	Omega-3 PUFA	Antioxidant activity	[229]
*Ulva lactuca*	Unsaturated fatty acid	Chemo-preventive	[230]
*Ulva armoricana* and *Solieria chordalis*	6:4n-3, 18:4n-3, 18:2n-3, 18:2n-6, 22:6n-3, 20:4n-6, and 20:5n-3	Antiproliferative activity	[231]
*C. racemosa*, *S. tenerrimum*, *C. indica* and *C. veravalnensis*	Palmitic acid (C16:0)-PUFAs	Antioxidant activity	[6]
*Spatoglossum asperum*	Mixture of fatty acids	Antifungal and nematicidal activities	[232]
*Enteromorpha linza*	Stearidonic acid (SA, C18:4 n-3) and gamma-linolenic acid (GLA, C18:3 n-6)	Antimicrobial activity	[233]
*Ceramium rubrum*	Mixture of fatty acids	Antibacterial and antifungal activities	[234]
*Kappaphycus alvarezii*	Levoglucosenone, 4-Pyridinemethanol, and Hexamethyl- cyclotrisiloxane	Antioxidant and antibacterial activity	[235]
*Palmaria palmata*, *Laminaria digitata*, *Saccharina latissima,* and *Ascophyllum nodosum*	Stearidonic acid, Eicosapentaenoic acid, Alpha-Linolenic acid, Docosahexaenoic acid, and Arachidonic acid	Anti-parasitic	[236]
*Ulva lactuca*, *Sargassum tenerrimum,* and *Laurencia obtusa*	Fatty acids and esters of fatty acids	Antifungal activity	[237]
*C. vagabunda*, *C. virgatum*, and *U. intestinalis*	Palmitic acid (C16:0), arachidonic acid (C20:4n-6), and oleic acid (C18:1ω-9cis)	Antimicrobial activity	[238]
*Tydemania expeditionis*	3(ζ)-hydroxy-octadeca-4(E),6(Z),15(Z)-trienoic acid (1) and 3(ζ)-hydroxy-hexadeca-4(E),6(Z)-dienoic acid	Antitumor activity	[239]

## Data Availability

The data used to support the findings of this study can be made available by the corresponding author upon request.

## Abbreviations

^1^O_2_	Singlet oxygen
ABTS	2,2′-azino-bis (3-ethylbenzothiazoline-6-sulfonic acid)
ACE I	Angiotensin-converting enzyme
AD	Alzheimer’s disease
AI	Atherogenicity index
ALS	Amyotrophic Lateral Sclerosis
ALT	Alanine aminotransferase
APC	Allophycocyanin
APTT	Activated partial thromboplastin time
AST	Aspartate aminotransferase
BHA	Butylated hydroxyanisole
BHT	Butylated hydroxytoluene
CFS	Chronic fatigue syndrome
CHIKV	Chikungunya virus
COVID-19	Coronavirus disease 2019
CVDs	Cardiovascular diseases
DENV-2	Dengue virus
DPPH	2,2-diphenyl-1-picrylhydrazyl
EFAs	Essential fatty acids
EPA	Eicosapentaenoic acid
FAs	Fatty acids
FCSPs	Fucose-containing sulfated polysaccharides
FFA	Free fatty acids
FRAP	Ferric-reducing ability of plasma
GLA	Gamma-linolenic acid
GPs	Glycoproteins
H_2_O_2_	Hydrogen peroxide
HFD	High-fat diet
HIV	Human immunodeficiency virus
hMPV	Human metapneumo virus
HSV-1	Herpes simplex virus type 1
HSV-2	Herpes simplex virus type 2
LDL	Low-density lipoprotein
LNA	Linolenic acid
MUFAs	Monosaturated fatty acids
O^2−^	Anion radicals
OH	Hydroxyl radicals
PC	Phycocyanin
PE	Phycoerythrin
PT	Prothrombin time
PUFAs	Polyunsaturated fatty acids
ROS	Reactive oxygen species
RSV	Respiratory syncytial virus
RVFV	Rift valley fever virus
SA	Stearidonic acid
SARS-CoV-2	Severe acute respiratory syndrome coronavirus 2
SFAs	Saturated fatty acids
TBA	Total bile acid
TBHQ	Tertiary butylhydroquinone
TI	Thrombogenicity index
TT	Thrombin time
UA	Uric acid
UAE	Ultrasonic-assisted extraction
UI	Unsaturation index
UV	Ultraviolet
WAT	White adipose tissue
